# Genomic Analysis of Glioblastoma Multiforme Reveals a Key Transcription Factor Signature Relevant to Prognosis and the Immune Processes

**DOI:** 10.3389/fonc.2021.657531

**Published:** 2021-04-27

**Authors:** Zhen-Hang Li, Yan-Lei Guan, Guo-Bin Zhang

**Affiliations:** ^1^ Department of Neurosurgery, Tianjin Huanhu Hospital, Tianjin, China; ^2^ Department of Neurosurgery, The First Hospital of China Medical University, Shenyang, China

**Keywords:** GBM, prognosis, bioinformatics, immunity, inflammation

## Abstract

**Introduction:**

Glioblastoma multiforme (GBM) develops through the accumulation of both genetic and expression alterations. Although many gene signatures have been developed as prognostic and predictive biomarkers, their robustness and functional aspects are less well characterized. The expression of most genes is regulated by transcription factors (TFs); therefore, we aimed to investigate a TF signature relevant to GBM prognosis.

**Methods:**

We used bioinformatic methods and data from public databases to establish four clusters of key TF genes, among which cluster 1, comprising 24 TFs, showed significant prognostic value. Further *in silico* functional analyses were applied to investigate the utility of the TF signature.

**Results:**

Different mutation and copy number variation patterns were observed between different risk score groups (based on the TF signature). *In silico* analyses suggested that the cases with relative high risk scores were involved in immune and inflammatory processes or pathways.

**Conclusion:**

The TF signature has significant prognostic value in different cohorts or subgroups of patients with GBM and could lead to the development immunotherapy for GBM.

## Introduction

Diffuse gliomas include lower-grade glioma (LGG, grade II and III) and the highly malignant glioblastoma multiforme (GBM, grade IV) (1). GBM is an aggressive and frequently diagnosed glioma. Under standard therapy, GBM has an average overall survival (OS) of 14.6 months and a 26.5% 2‑year survival rate (2). The “integrated” phenotypic and genotypic parameters for central nervous system (CNS) tumor classification were introduced in the 2016 World Health Organization (WHO) Classification of Tumors of the Central Nervous System (3), which emphasizes the molecular impact on the tumorigenesis and prognosis of glioma. The clinical hallmarks of the poor prognosis of GBM comprise inevitable recurrence, limited therapeutic response, and aggressive growth (4). The development of molecularly targeted methods has resulted in a paradigm shift in the diagnosis and treatment of cancers. Informed therapeutic choices are increasingly made by combining histology with molecular analysis (5, 6).

Glioma, especially GBM, has high heterogeneity, which originates from complex interactions between developmental and genetic factors. To date, based on transcriptomic classification, GBM has been divided into classical (CL), neural (NE), proneural (PN), and mesenchymal (ME) subtypes (7). Increasing research has attempted to identify prognostic molecular markers. However, little attention has paid to related transcription factors (TFs) as markers. Therefore, it would be clinically significant to identify a TF signature representing a tumor’s intrinsic characteristics and heterogeneity to predict patient outcome.

The control of eukaryotic gene expression involves a combination of regulatory signals exerted by a variety of factors (8). The integration of regulatory elements, such as microRNAs, epigenetic markers, specific transcription factors and their cofactors, and other input signals, allows the coordination of gene expression patterns in a spatiotemporal context‑dependent manner (9). In particular, TF control of gene expression is a highly conserved mechanism by which signals are integrated in key regulatory pathways, and their study permits the identification of the TF-controlled genes and their associated regulatory mechanisms. “Cooperative” TFs participate in multi-protein complexes that often recruit further TFs or cofactors to fine‑tune their regulatory abilities (10). In protein interaction networks, the distances between cooperative TFs are shorter and more clustered than would be expected by chance (11). In addition, TFs promote regulatory activities in basic eukaryotic processes, such as the cell cycle (12), cell differentiation (13), immunity (14), and malignant transformation (15, 16). Emerging evidence suggests that dysregulation of immunity and cancer initiation are closely correlated (15, 16). However, the complex roles of cooperative TFs in the context of glioma are unclear.

The current study aimed to develop a key signature TF gene set that correlated with patient prognosis. To achieve this, a supervised approach associated with clinical covariates was carried out. This resulted in a combined analysis that identified a robust immunity and inflammation‑related TF gene set and the establishment of a risk score system. Further bioinformatic analyses revealed that the risk score had good prognostic value in stratifying patients and was associated with different mutation or copy number variation (CNV) patterns.

## Materials and Methods

### Datasets

Normalized whole genome mRNA expression microarray data and associated clinical date were obtained from The Cancer Genome Atlas (TCGA) GBM dataset (7) (http://cancergenome.nih.gov/) as the training cohort. As validation cohorts, three datasets were obtained: GSE16011 from the Gene Expression Omnibus database (http://www.ncbi.nlm.nih.gov/geo/query/acc.cgi?acc=GSE16011) (17), whole genome mRNA expression RNA-seq data from the Chinese Glioma Genome Atlas (CGGA) database (http://www.cgga.org.cn) (18), and RNA‑seq data integrated with CNV and somatic mutation data from the TCGA glioma (lower-grade glioma, LGG and GBM) project (19). The training dataset comprised 525 cases (GSE16011 n = 155, CGGA RNA-seq n = 117 GBM samples; TCGA RNA-seq n = 663 glioma samples (153 GBMs and 510 LGGs)). Patients’ characteristics are summarized in **Table S1**.

### Statistical Analysis

The time interval from the diagnosis date to death or last follow-up defined overall survival (OS). The differences in prognosis for patients with high or low expression of a certain gene or risk score (compared with the median value) were calculated using the Kaplan–Meier method, together with a two-sided log-rank test, in the “survival” package of the R software (version 4.0.3 for Windows). We also used the “survival” package to perform univariate and multivariate COX regression analysis. Principle component analysis (PCA) of the genes was also carried out in R. To compare the numerical values between two groups, we used a two‑tailed Student’s t-test. To analyze the differences of the means among groups, we used analysis of variance (ANOVA). To compare the frequencies between groups, we used Fisher’s exact test and the Chi‑squared test. For receiver operating characteristic (ROC) curve analysis and comparisons between factors, we used the package “pROC” in R. To combine two factors, we used fitting of a generalized linear model. Area Under the Receiver Operating Characteristic Curve (AUC) estimation was used to evaluate prediction performance. In the ROC analysis, we excluded those patients who were not censored at the last follow-up and whose disease durations were less than the mean OS. Associations between two variables were performed using Pearson correlation (r) analysis. Statistical significance was accepted at P <0.05.

### Bioinformatic Analysis

Differentially expressed genes (DEGs) were identified using the “Limma” package of R according to a false discovery rate (FDR) threshold of less than 0.05. In R, the “ConsensusClusterPlus” package was used to cluster genes into different subgroups (20). STRING was used for protein‑protein interaction analysis (21). Relevant biological implications were investigated using the “TCGAbiolinks” package of R (22). Gene set enrichment analysis (GSEA) (23) was used to further verify the biological phenotypes. The gene set variation analysis (GSVA) package of R (24) was used to construct the immune cell gene sets and single sample GSEA (ssGSEA) enriched gene sets, based on the summarized gene list of immune cells described by Gabriela et al. (25).

## Results

### Selection of Transcription Factors and Their Role in Different Categories of Glioma

**Figure 1:**
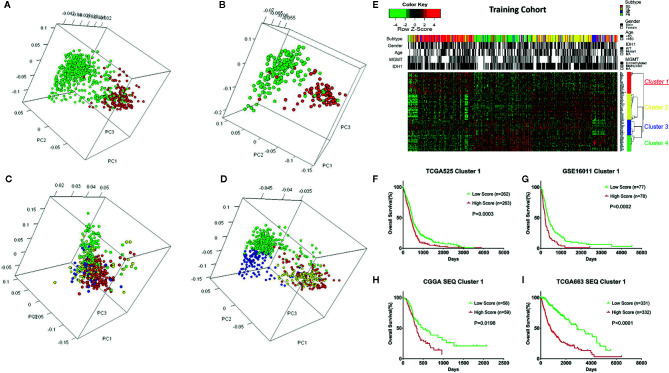
Overview of the transcription factors (TFs) used to stratify different grades, prognosis, and subtypes of glioma, and the selection of the TF signature with its prognostic value across cohorts. **(A)** Principle component analysis (PCA) indicating that the TFs could distinguish LGG from GBM in the TCGA RNA-seq cohort. Green: LGG; Red: GBM. **(B)** PCA indicating that the TFs could distinguish long-term survival from short-term survival in the TCGA RNA-seq cohort. Green: OS > 3 years; Red: OS < 1 year. **(C)** PCA indicating that the TFs could distinguish different transcriptional subtypes of GBM in the training cohort. Green: PN; Blue: NE; Yellow: CL; Red: ME. **(D)** PCA indicating that the TFs could distinguish different transcriptional subtypes of glioma in the TCGA RNA-seq cohort. Green: PN; Blue: NE; Yellow: CL; Red: ME. **(E)** Heatmap depicting the Z-scored expression values and the clustering of 88 key TF genes in the training cohort. Columns represent each sample and are labeled with their clinical characteristics, rows represent genes and were clustered into four groups. Cluster 1 represents the signature. **(F)** Kaplan–Meier survival analysis based on the median cutoff value of the risk score developed using the cluster 1 gene set in the training cohort. **(G)** Kaplan–Meier survival analysis based on the median cutoff value of the risk score developed using the cluster 1 gene set in the GSE16011 cohort. **(H)** Kaplan–Meier survival analysis based on the median cutoff value of the risk score developed using the cluster 1 gene set in the CGGA RNA-seq cohort. **(I)** Kaplan–Meier survival analysis based on the median cutoff value of the risk score developed using cluster 1 gene set in the TCGA RNA-seq cohort. LGG, Lower grade glioma; Mutant, IDH1 mutant; WT, IDH1 wild type; NE, Neural; PN, Pro-neural; CL, Classical; ME, Mesenchymal; NA, Not acquired.

A total of 525 patients with GBM with mRNA expression and clinical data were obtained as the training cohort from the TCGA database. 1666 TFs were confirmed from the whole gene expression data. To investigate the role TFs in regulating the biological behavior and clinical outcome of glioma, PCA was carried out using the TFs of either cohort. The TFs could distinguish LGG from GBM (**Figure 1A**) and long-term survival from short-term survival (**Figure 1B**) in the TCGA RNA‑seq cohort. Furthermore, the TFs showed different patterns of distribution in the four transcriptional subtypes (CL, NE, PN, and ME) in either the training (**Figure 1C**) or TCGA RNA-seq (**Figure 1D**) cohorts. Median absolute deviation (MAD) was calculated for each of the 1666 TFs in the training cohort from 525 tumor samples to further select genes with high heterogeneity. The resulting 88 TFs with an MAD >1.0 were thus defined as key regulators.

### Clustering of The Key TFs and The Prognostic Value of Clusters

Different TFs have different functions in regulating glioma oncogenesis and progression. Therefore, we performed consensus clustering of the 88 TFs to cluster them into different groups with similar expression profiles. Four clusters of TFs were defined in the training cohort (**Figure 1E** and **Supplementary Figures S1, S2A-D**). Thereafter, we calculated a score for each cluster using the ssGSEA method. Of the four cluster scores, only the score for dichotomized cluster 1 (risk signature: *AHR*, *ATF3*, *BLNK*, *CEBPA*, *EGR2*, *FAS*, *FHL2*, *FOS*, *HCK*, *ID1*, *IQCG*, *MAFB*, *MYLK*, *MYO1B*, *NR2F2*, *PDLIM1*, *PDLIM4*, *PLK2*, *PRRX1*, *SAMSN1*, *SLA*, *SNAI2*, *TNFRSF11B*, *and TWIST1*) showed significant prognostic value in the training cohort (**Figure 1F**, P = 0.0003). To confirm whether the genes in the TF signature were “cooperative”, Pearson’s correlation value was calculated between all pairs of genes (mean r = 0.241, **Supplementary Figure S2E**) and protein-protein interaction (PPI) analysis was performed (**Supplementary Figure S2F**). To validate the TF risk signature in other populations, we calculated the risk score for each patient in the validation cohorts using the same method. Patients were classified into high or low risk groups in comparison with the median risk score. As expected, the survival curves showed a greater reduction in OS for the high risk patients than the low risk ones (**Figures 1G–I**).

### Risk Score Distribution and Prognostic Value among Glioma Subgroups

Clinicopathological factors, such as transcriptional subtypes, the methylation status of *MGMT* (encoding O-6-methylguanine-DNA methyltransferase), the mutation status of *IDH1* (encoding isocitrate dehydrogenase (NADP(+)) 1), grade, sex, and age, were used to stratify the patients in the training cohort. Patients with male sex (P = 0.027), *IDH1* wild-type (P = 3.1e-06), and unmethylated *MGMT* status (P = 0.029) demonstrated higher risk scores, whereas age (P = 0.82) had almost no effect on the distribution of the risk score. For the transcriptional subtypes (P < 2.2e-16, ANOVA), the ME subtype had the highest risk score (median 0.321) and the PN subtype had the lowest risk score (median 0.172), whereas the NE and CL subtypes had intermediate risk scores (**Supplementary Figure S3A**). There were only two overlapping genes (*FHL2* and *MAFB* in the ME subtype) between the TF signature and the transcriptional subtype genes. The distribution of the risk score among subgroups in the validation cohorts is shown in **Supplementary Figure S4**, which showed good correspondence with the distribution pattern in the training cohort. Notably, the risk score increased with grade progression in the TCGA RNA-seq cohort. To validate the different risk score patterns in the transcriptional subtypes, we queried the genes representing the highest and lowest risk score subtypes (mesenchymal and proneural gene sets) and sorted the expression profile of these genes according to increased risk score (**Supplementary Figure S3B**). This analysis showed that the expression value of mesenchymal genes was higher with increased risk score, while the opposite pattern was observed for proneural genes. Furthermore, Pearson correlation analysis was conducted with key genes in either gene set. *CHI3L1*, also known as *YKL‑40* (r = 0.520, P <0.001; **Supplementary Figure S3C**) and *TGFB1* (r = 0.520, P <0.001; **Supplementary Figure S3D**) correlated positively with the risk score, while *OLIG2* (r = −0.450, P < 0.001; **Supplementary Figure S3E**) and *DLL3* (r = −0.402, P < 0.001; **Supplementary Figure S3F**) showed a negative correlation.

**Figure 2:**
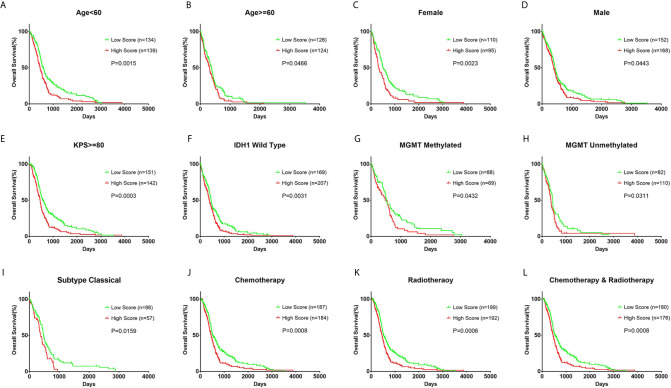
Prognostic value of the risk score in different clinicopathological subgroups. **(A, B)** Kaplan–Meier survival analysis based on the median cutoff value of the risk score developed using the cluster 1 gene set in the training cohort in different age subgroups. **(C, D)** Kaplan–Meier survival analysis in different sex subgroups. **(E)** Kaplan–Meier survival analysis in KPS ≥ 80 patient subgroup. **(F)** Kaplan–Meier survival analysis in *IDH1* wild-type patient subgroup. **(G, H)** Kaplan–Meier survival analysis in different MGMT methylation status subgroups. **(I)** Kaplan–Meier survival analysis in the classical subtype patient subgroup. **(J–L)** Kaplan–Meier survival analysis based on different patient subgroups that underwent chemotherapy, radiotherapy, or combination therapy. KPS: Karnofsky Performance Status.

In each subgroup, the patients were assigned to either a high or low risk supergroup on the basis of the median cut-off of the risk score in the training cohort. To determine the prognostic value, the dichotomized risk score of the whole cohort was applied to all the subgroups. The results were almost universal among most of the subgroups (**Figure 2**), in that a high risk score correlated markedly with poor prognosis and a low risk sore was associated with better prognosis. After adjusting for other clinical covariates, the risk score was identified as an independent prognostic factor using univariate and multivariate Cox regression analyses (**Table 1**). Furthermore, the risk score was applied in the TCGA RNA-seq cohort for ROC analysis to explore the sensitivity and specificity of the risk score compared with other covariates, providing the AUC of disease status (GBM *vs*. LGG, AUC = 0.6953), *IDH1* mutation status (wild-type *vs*. mutant, AUC = 0.7711), *MGMT* methylation status (unmethylated *vs*. methylated, AUC=0.6488), and the risk score (as a continuous variable, AUC = 0.7490, **Supplementary Figure S5**). The risk score outperformed disease status (P = 0.0192) and *MGMT* methylation status (P = 0.0001), but showed no significant difference compared with the *IDH1* mutation status (P = 0.2954). A significantly higher AUC (combined, AUC = 0.8072) was achieved when fitting of a generalized linear model was applied to the risk score (P < 0.0001) and *IDH1* mutation status (P = 0.0060).

**Table 1:** COX regression analysis of the risk score based on the TF signature and other covariates in GBM.

Variables	TCGA Microarray Univariate		TCGA Microarray Multivariate	
	HR	P	HR	P
Signature (High *vs*. Low)	1.41	**0.0003**	1.42	**0.0260**
Age (≥ 60 *vs*. < 60)	1.85	**<0.0001**	1.35	0.0681
Sex (Male *vs*. Female)	1.16	0.1223	–	–
KPS (<80 *vs*. ≥80)	2.18	**<0.0001**	1.37	0.0926
*IDH1* (Wild-type *vs*. Mutant)	2.86	**<0.0001**	2.00	**0.0259**
*MGMT* (Unmethylated *vs*. Methylated)	1.38	**0.0086**	1.09	0.5941
Chemotherapy (Yes *vs*. No)	0.41	**<0.0001**	0.74	0.2729
Radiotherapy (Yes *vs*. No)	0.35	**<0.0001**	0.34	**<0.0001**

HR, hazard ratio; Univariate, Univariate Cox Regression; Multivariate, Multivariate Cox Regression; Bolded values indicates statistically significant.

### Distinct Patterns of CNVs an Somatic Mutations were Associated with the Risk Score

**Figure 3:**
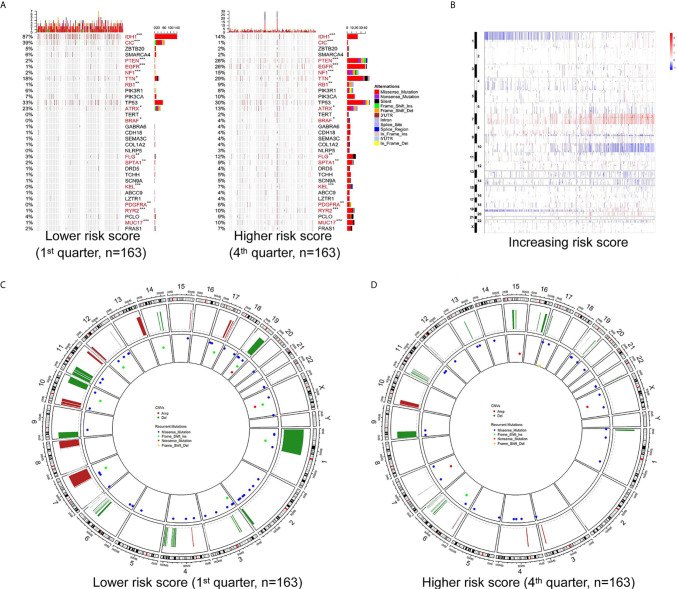
Different mutation and copy number variation pattern of the risk score. **(A)** Summary of well-known individual regulators of glioma from the lower and higher risk score samples from the TCGA RNA-seq cohort. Columns are sorted by samples with increasing risk score. Top histogram, sum of mutations in each sample category indicated by the legend; Right histogram, sum of mutations in each gene indicated by the legend. **(B)** The overall copy number variation (CNV) profile in order of increasing risk score in the TCGA RNA-seq cohort. **(C, D)** A distinct recurrent CNV and mutation profile is observed between gliomas with a lower and higher risk score in the TCGA RNA-seq cohort. *P < 0.05; **P < 0.01; ***P < 0.001.

To further determine the effect of the risk score at the DNA level, the TCGA RNA-seq data, with available CNV and somatic mutation information, which was more comprehensive than the training microarray cohort, were analyzed. Using the increasing risk score as the basis, we divided the cases into four subgroups that were more representative for intergroup comparison. We then analyzed the mutation status of known glioma regulators (**Figure 3A**). Frequent mutations in *IDH1* (p < 0.001), *CIC* (p <0.001), and ATRX (p = 0.029) showed significant enrichment in cases with a lower risk score. *PTEN* (p < 0.001), *EGFR* (p < 0.001), *NF1* (p < 0.001), *PDGFRA* (p = 0.002), *RB1* (p = 0.002), and *BRAF* (p = 0.015) mutations were enriched significantly in cases with a higher risk score. In addition, significantly different mutation frequencies of *FLG*, *RYR2*, *TTN*, *SPTA1*, *MUC17*, and *KEL* were attributed to various risk score subgroups. However, existing studies have barely explored their roles in glioma.

Investigation of the CNV data between the high and low risk score groups revealed distinct chromosomal alteration patterns. With decreasing risk score, the incidence of the 1p/19q codeletion (a genomic hallmark of oligodendroglioma) increased. The frequency of the GBM representative event comprising Chr 7 amplification paired with Chr 10 loss increased in the high risk score group (**Figure 3B**). Additional CNVs comprising frequently deleted genomic regions were 9p21.3, which encompasses *CDKN2A* and *CDKN2B* (mean deletion, *CDKN2A* −0.162 1^st^ quarter *vs*. −0.682 4^th^ quarter, p <0.001; *CDKN2B* −0.162 1^st^ quarter *vs*. -0.659 4^th^ quarter, p <0.001), and 10q23.3 encompassing PTEN (−0.067 1^st^ quarter *vs*. −0.574 4^th^ quarter, p <0.001). While 7p11.2, which encompasses *EGFR* (mean amplification, 0.087 1^st^ quarter *vs*. 1.732 4^th^ quarter, p <0.001), *PDGFRA* (4q12; -0.038 1^st^ quarter *vs*. 0.422 4^th^ quarter, p <0.001), *CDK4* (12q14.1; 0.039 1^st^ quarter *vs*. 0.483 4^th^ quarter, p <0.001), and mouse double minute 2 homolog or 4 homolog (*MDM2*/*MDM4*; 12q15/1q32.1; *MDM2* −0.016 1^st^ quarter *vs*. 0.171 4^th^ quarter, p = 0.006; *MDM4* 0.021 1^st^ quarter *vs*. 0.387 4^th^ quarter, p < 0.001) were amplified frequently, with a higher risk score (**Figures 3B–D**).

### High Risk Score GBM Exhibited an Immunity and Inflammatory Enriched Phenotype

**Figure 4:**
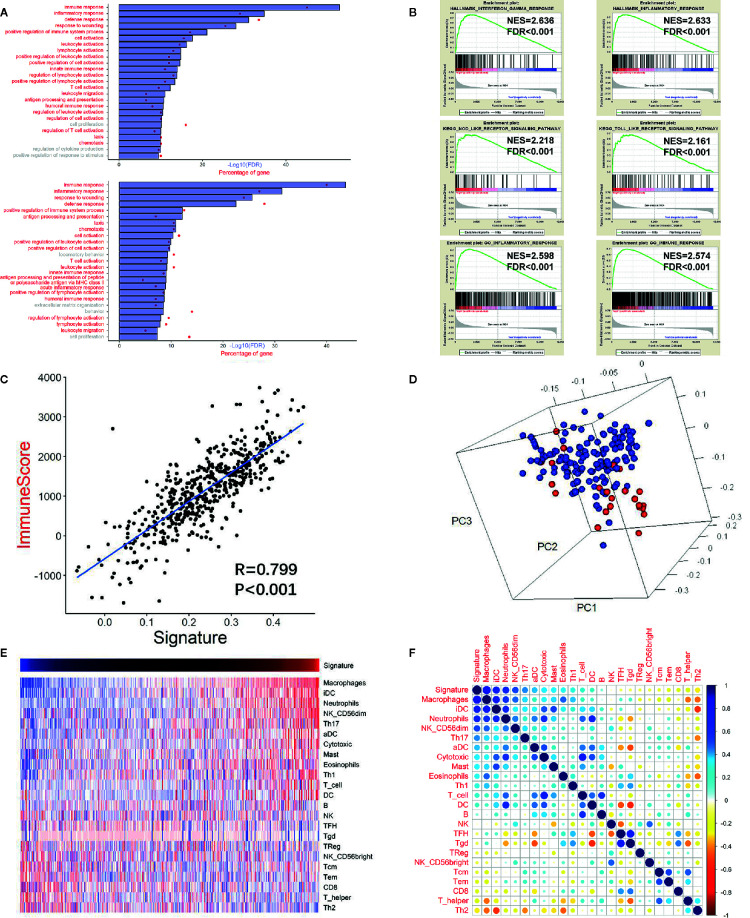
High risk score GBM exhibited immune and inflammatory enriched phenotype. **(A)** Upper chart: Top 25 GO terms enriched by genes that correlated highly (r > 0.4) with the risk score, suggested that they were highly enriched in immunity, inflammation, and related processes (red). Lower chart: Top 25 GO terms enriched by the upregulated DEGs (at the FDR of 0.05 and the lowest log fold change of 0.5) analyzed between the high risk score group and the low risk score group. **(B)** GSEA analysis reveals that the risk score was associated with processes or pathways closely associated with immunity, inflammation, and related processes. **(C)** Pearson correlation analysis between the risk score (signature) and the immune score of the training cohort. **(D)** PCA showing that the genes used to construct the risk score were highly associated with the genes used to construct the immune score gene set. Red: genes of the risk score gene set; Blue: genes of the immune score gene set. **(E)** Heatmap showing the relationship between the risk score (signature) and the ssGSEA score of each immune cell. Red: high score; Blue: low score. **(F)** Pearson correlation analysis between the risk score and the ssGSEA score of each immune cell. The vertical bar indicates the r value.

GO analysis was carried out to assess the functional features associated with the prognostic value and different patterns of CNVs and somatic mutations depending on the risk score, using the Pearson correlation score (r) calculated for each gene in the training cohort. Using 779 genes whose expression correlated positively with the risk score (r > 0.4), the GO analysis revealed high enrichment for immunity, inflammation, and their related functions. For validation, DEGs analysis was performed based on the high or low risk scores in the training cohort. This identified that 751 genes were upregulated in the high risk score group (FDR <0.05 and a lowest log-fold change of 0.5), which were then subjected to GO analysis (**Figure 4A**). The GO results for the positively correlated genes and the DEGs both identified enrichment for immunity, inflammation, and their related functions. Meanwhile, cell proliferation was also found in the GO results in both panels. The same method was applied to the other three validation cohorts, and similar results (immune response and inflammatory response being the top two GO annotations) were obtained (**Supplementary Figure S6**).

Next, we performed GSEA of the risk score, which demonstrated an association between the risk score and pathways and processes that are closely related to inflammation and immunity. Hallmark interferon gamma response, Hallmark inflammatory response, KEGG NOD-like receptor signaling pathway, KEGG TOLL-like receptor signaling pathway, GO inflammatory response, and GO immune response were among the top enriched GSEA terms in the high risk score group (**Figure 4B**).

GO and GSEA analyses based on the other three TF gene sets indicated that the cluster 2 gene set was associated with neuron development, the cluster 3 gene set was associated with the cell cycle, and the cluster 4 gene set was associated with metabolic and neuron morphogenesis (**Supplementary Figure S7**). The failure of the three gene sets’ ssGESA scores to achieve a significant prognostic value suggested that the related biological functions might be universal processes regulating glioma malignancy.

### Association between The Immune Score and The Risk Score

The high correlation between the immunity and inflammatory processes and pathways and the risk score suggested that tumor and non-tumor fractions (e.g., immune cells) were present in glioma tumors. Therefore, to infer the tumor compartment, a recently developed universal algorithm based on transcriptomic expression data was used. The algorithm can quantify the tumor cell content in the tissue and estimate immune cell infiltration. Thus, for each sample, an immune score was calculated using the ESTIMATE algorithm. A high correlation was observed between the risk score and the immune score (r = 0.799, P < 0.001, **Figure 4C**). Subsequently, PCA was carried out based on the genes constructing the risk score and the genes constructing the immune score (**Figure 4D**). The result confirmed that the genes were highly associated with the immune score gene set. Moreover, many immune checkpoint genes (*CLL2*, *CD4*, *CD80*, *CD86*, *CXCR4*, *ICOSLG*, *IL6*, *IL10*, *LGALS9*, *PDCD1LG2*, *TGFB1*, *TNFRSF9*, *and TNFSF4*) were also found to be more highly expressed in the high risk score group than in the low risk score group in the training cohort (**Supplementary Figure S8**).

### Correlation of The Risk Score and Immune Cells

There was a high correlation between the risk score and immunity according to the GO and GSEA analyses. Therefore, we investigated which immune cells were important for the immune processes in the glioma microenvironment. Thus, ssGSEA analysis was performed based on a gene list of immune cells summarized by Gabriela et al. (25). Pearson correlation analysis was performed between immune cell enrichment scores and the risk score. There was a significantly high correlation between the survival of pernicious immune cells (26) and the risk score: Macrophage cells (r = 0.817, P <0.001) and neutrophils (r = 0.582, P <0.001) (**Figures 4E, F** and **Supplementary Table S2**). However, the enrichment score of neutrophils could not achieve a significant prognostic impact on overall survival (P=0.787; log rank test) in the training cohort. The risk score also correlated significantly with other immune cells, including T follicular helper (TFH) B cells (r = −0.083, P = 0.059), B cells (r = 0.078, P = 0.072), and natural killer cells (r = −0.027, P = 0.537). In summary, in the tumor microenvironment, immune cell enrichment correlated highly with the risk score.

## Discussion

The immune microenvironment in glioma is not well understood, such that interactions between the host immune system and the tumor, as well as the molecular pathogenesis of glioma, await better characterization. Personalized drugs, including multimodal immunotherapy, represent a reasonable, optimal, and flexible method to induce long-term tumor control (27). The identification of predictive and prognostic biomarkers for glioma could help to optimize therapy decisions. In this study, we analyzed the gene expression profiles from 525 GBM tumors and identified a robust TF gene signature that is relevant to immune-related processes. The signature- based risk score exerted its prognostic stratifying ability either in the training or validation cohorts, and could distinguish gliomas with different mutations or CNV patterns. Notably, a positive correlation was observed between the risk score and mesenchymal genes of glioma, while a negative correlation was observed between the risk score and proneural genes. Moreover, the risk score demonstrated high correlation with the immune score. In accordance with our risk score, mesenchymal glioma demonstrated worst prognosis while proneural glioma had the best prognosis (7). Many kinds of cancers that undergo epithelial-to-mesenchymal transition (EMT) show significant enrichment of multiple immune targets (28, 29), which further validated the high correlation with immunity in the glioma scenario.

Genes constituting our signature could be regarded as alternative targets, alone or in combination, according to their regulatory nature and prognostic significance. For instance, the TDO-AHR pathway is active in human brain tumors, in which it could suppress anti-tumor immune responses and was associated with malignant progression and poor survival (30). The FAS-FAS ligand system in human brain tumors was shown to be involved not only in apoptotic processes, but also in the promotion of angiogenic and proinflammatory responses (31). FHL2 interacts with EGFR and EGFRvIII to increase their levels and promotes glioma growth (32). ID1 regulates multiple tumor-promoting pathways, such as invasiveness and self-renewal in glioblastoma (33). PRRX1 could potentiate glioma-initiating cells *via* DRD2-mediated ERK and AKT activation (34). SNAIL2 and TWIST1 act as inducers for cell‑invasiveness and EMT in GBM (35, 36). Furthermore, multiple targetable immune checkpoint genes were expressed at higher levels in the high risk score group. For example, increased expression of CCL2 might activate neutrophils through the IL6-STAT3-PDL1 signaling cascade (37). B7 and CD28 family cell surface molecules (CD80 and CD86) have important functions in T-cell tolerance and activation (38). ICOSLG, a member of the B7 family of costimulatory molecules related to CD80/CD86, regulates CD4 and CD8 T-cell responses *via* interaction with its receptor, ICOS, on activated T cells (39). PDCD1LG2 (PD-L2)-specific T (CD4 or CD8) cells support anti-cancer immunity directly by killing their target cells (38). Gliomas result in the upregulation of B7-H1 expression in tumor-infiltrative macrophages and circulating monocytes by modulating autocrine and paracrine IL10 signaling, which produces an immunosuppressive phenotype (40). Gagner et al. suggested that inhibition of CXCR4 regulated tumoral, stem cell, and immune mechanisms *via* adjunctive CXCR4 antagonists, which might help to overcome antiangiogenic therapy resistance, benefiting patients with GBM (41). Liu et al. suggested that the LGALS9-Tim-3 pathway might be critical in the immuno-evasion of glioma and might be a potent target for immunotherapy in patients with glioma (42). It was reported that SD‑208, a TGFB1 inhibitor, could enhance the immunogenicity and inhibit the growth and invasiveness of murine and human glioma cells (43). In multiple immune cell subsets, TNFRSF9 (CD137) provides a costimulatory signal, suggesting that combination therapy comprising CD137 antibodies with therapeutic antibodies and/or vaccination might improve cancer treatment (42). TNFSF4 (OX40L, as known as CD134) has been reported to regulate the T-cell response, leading to a study of OX40L inhibition combined with other checkpoint blockades (44). Microarrays for gene expression profiling and other quantitative methods (such as RNA-seq) are being used to facilitate the targeting signature gene expression in GBM. The alternative expression patterns of these genes might facilitate future drug design.

## Conclusion

The TF signature has significant prognostic value in different cohorts or subgroups of patients with GBM. The analysis of TF genes might allow the systematic prioritization of different types of immunotherapeutic strategies. The TF signature could be used to identify those patients who might respond to a certain strategy, thus allowing selective enrichment of potential responders during small-scale or early clinical trials.

## Data Availability Statement

The original contributions presented in the study are included in the article/**Supplementary Material**. Further inquiries can be directed to the corresponding author.

## Author Contributions

Z-HL conceived the study, interpreted the results, and wrote the manuscript. Y-LG and G-BZ supervised the acquisition of the data. G-BZ revised the manuscript. All authors contributed to the article and approved the submitted version.

## Funding

This study was supported by Tianjin Key Clinical Discipline Construction Project.

## Conflict of Interest

The authors declare that the research was conducted in the absence of any commercial or financial relationships that could be construed as a potential conflict of interest.
