# Supplementary material for: Genomic Analysis of Glioblastoma Multiforme Reveals a Key Transcription Factor Signature Relevant to Prognosis and the Immune Processes
Source: Front Oncol. 2021 Apr 27;11:657531. doi: 10.3389/fonc.2021.657531 (PMC8112242; doi:10.3389/fonc.2021.657531)
Supplement: Supplementary file 1 [file DataSheet_1.doc]

**
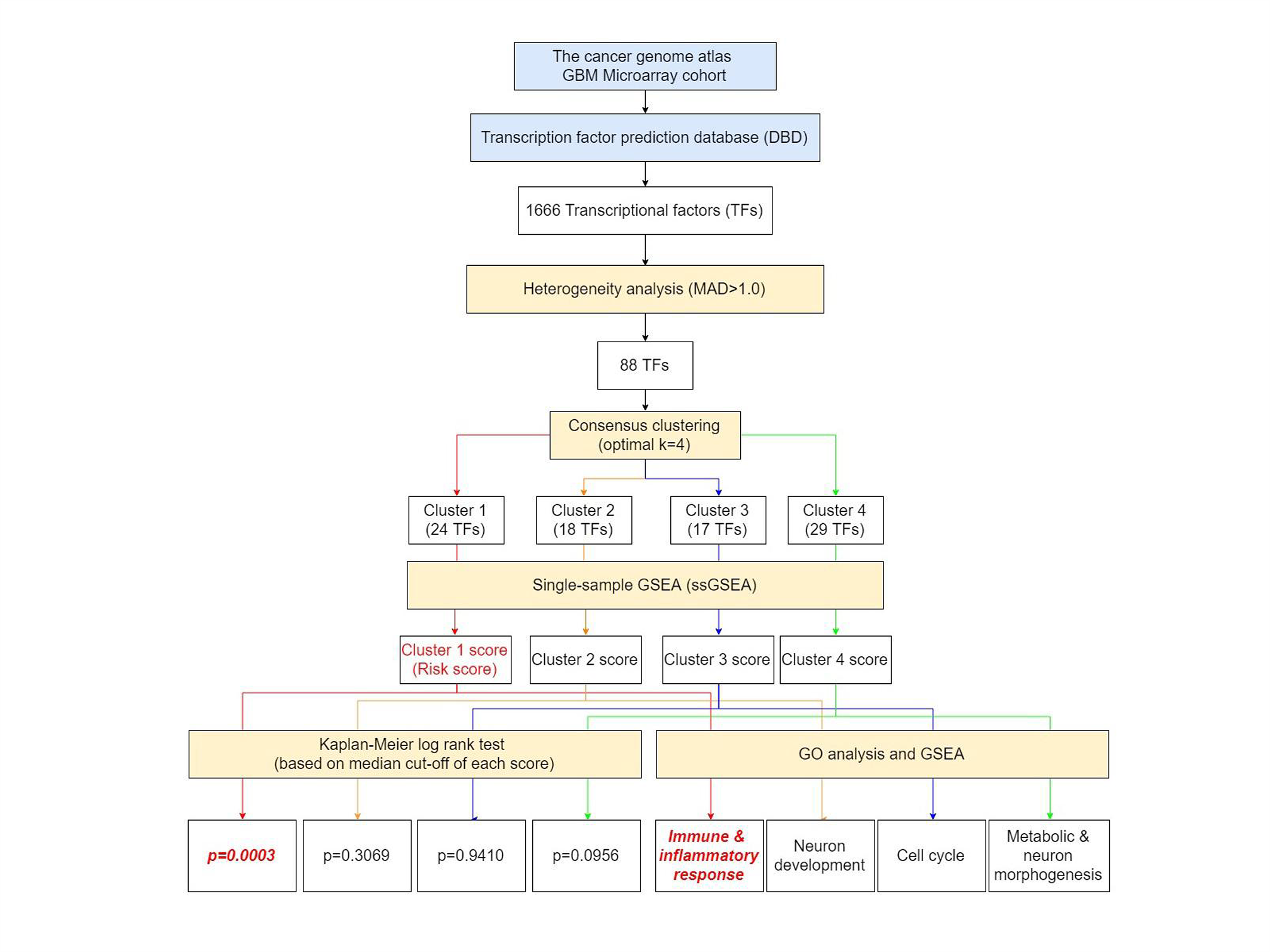
** **Supplementary Figure 1.** **Work-flow of the selection of the key TF gene sets and the relevant functional annotation of the gene sets.**


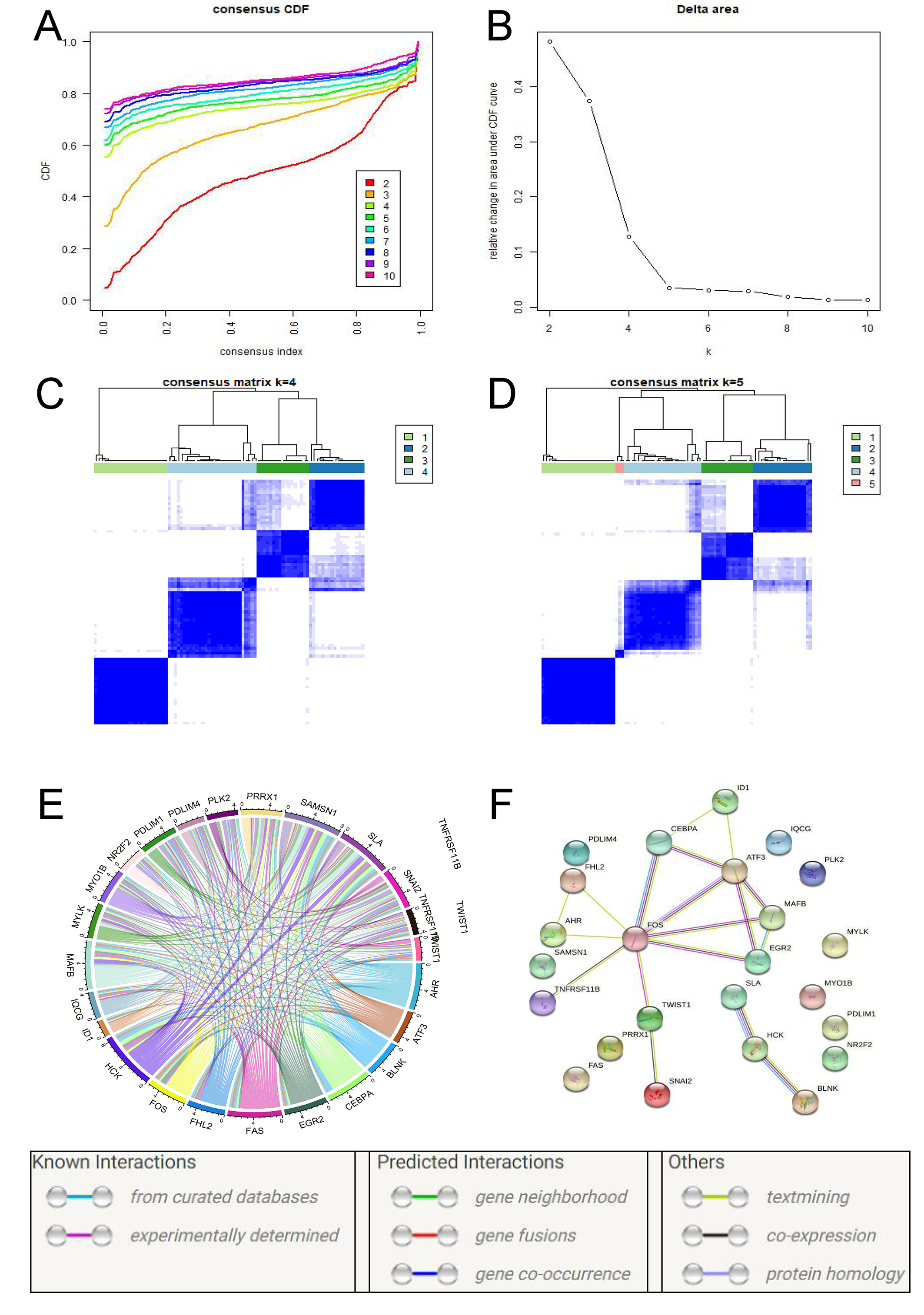


**Supplementary Figure 2.** **Selection of the best cluster and the mutual correlation of the signature genes. (A-D)** Best k selection of the key TF genes. From **(A)** and **(B)** we indicated that the best k might be **(C)** k=4 or **(D)** k=5. When k=5, there were just 3 genes different from k=4. However, the results of k=4 or k=5 did not affect the genes of the signature TF gene set. Further, cluster 1 gene set (signature) showed little correlation with other genes. **(E)** Association (Pearson correlation) among genes in the signature. Stripe width indicated the r value. **(F)** Protein-protein interaction (PPI) analysis among the genes of the signature.


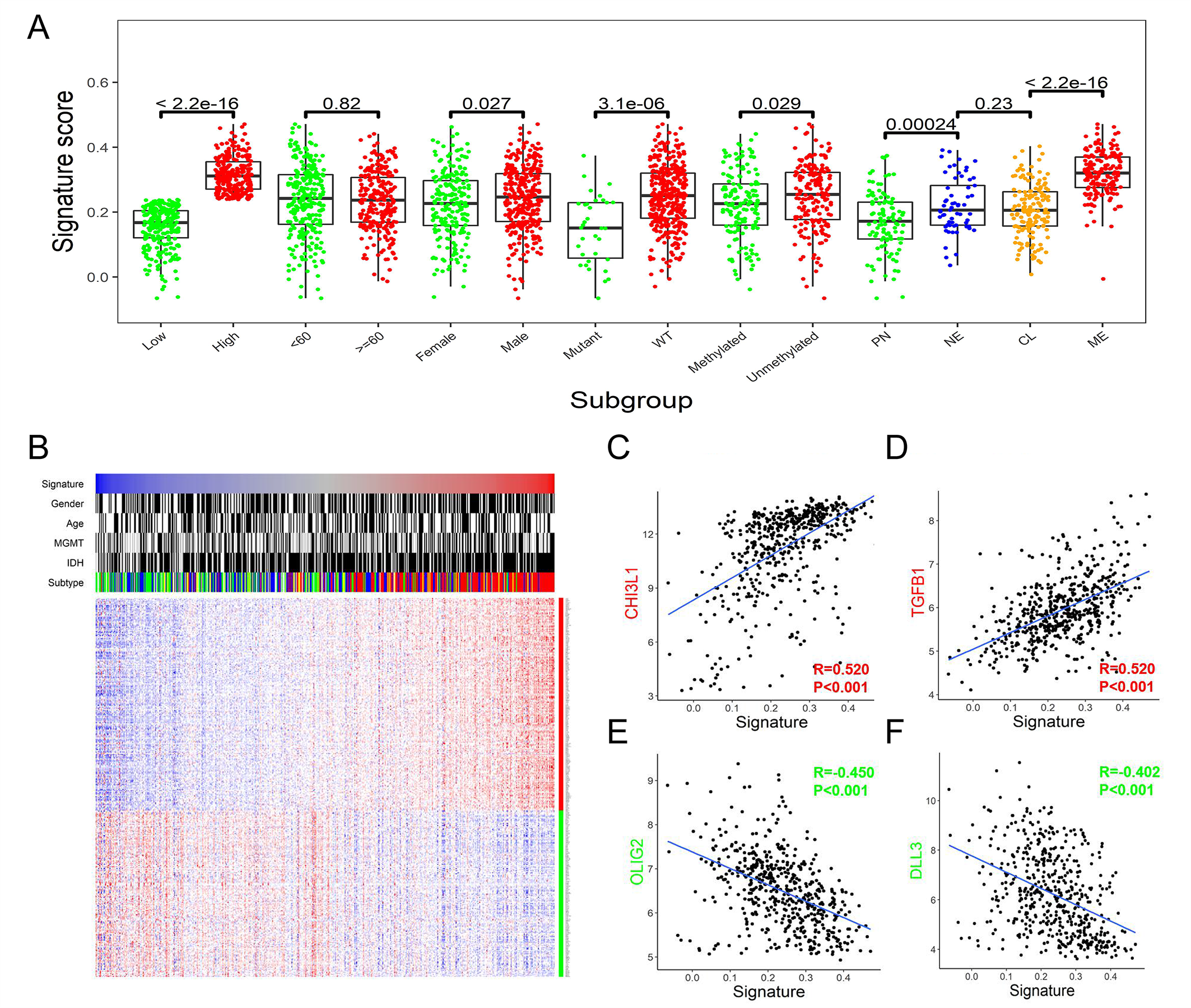


**Supplementary Figure 3. Distribution of the risk score in different subgroups, and the relationship between the risk score and transcriptional subtype genes.** **(A)** Distribution of the risk score among subgroups in the training cohort. **(B)** Heatmap depicted the Z-scored expression value of the genes representative of mesenchymal (red vertical bar) and proneural (green vertical bar) subtype in the training cohort. Columns represented each sample and were labeled with their clinical characters, rows represented genes. **(C-F)** Pearson correlation between the risk score and the well-known genes representative of mesenchymal (red) and proneural (green) subtype in the training cohort. Mutant: IDH1 mutant, WT: IDH1 wild type; NE: Neural, PN: Pro-neural, CL: Classical, ME: Mesenchymal.


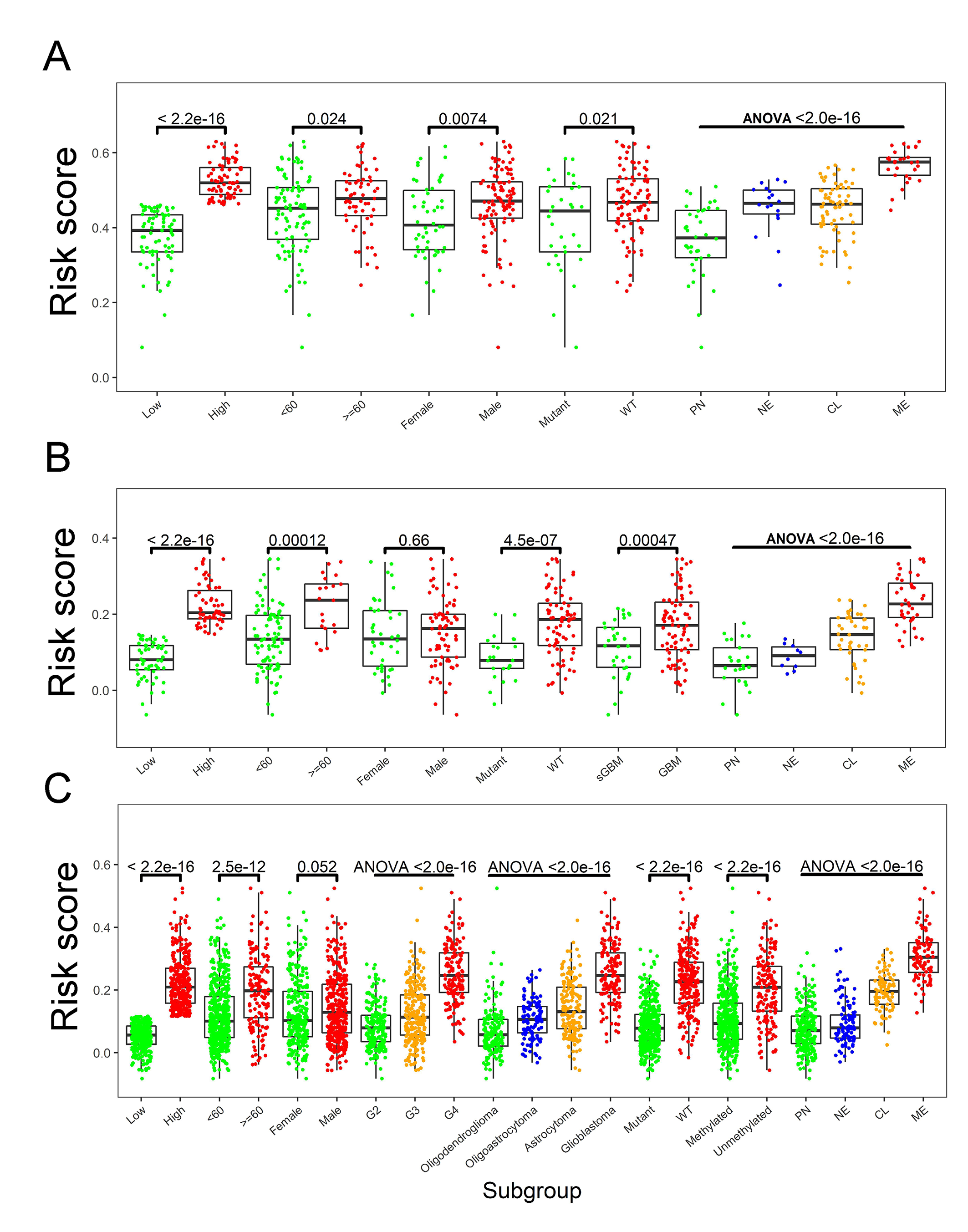


**Supplementary Figure 4. Distribution of the risk score in validation cohorts. (A)** Distribution of the risk score among subgroups of GBM in the validation cohort GSE16011. **(B)** Distribution of the risk score among subgroups of GBM in the validation cohort CGGA RNA-seq. **(C)** Distribution of the risk score among subgroups of glioma in the validation cohort TCGA RNA-seq.

G2: Grade II, G3: Grade III, G4: Grade IV; sGBM: Secondary GBM; Mutant: IDH1 mutant, WT: IDH1 wild type; NE: Neural, PN: Pro-neural, CL: Classical, ME: Mesenchymal.


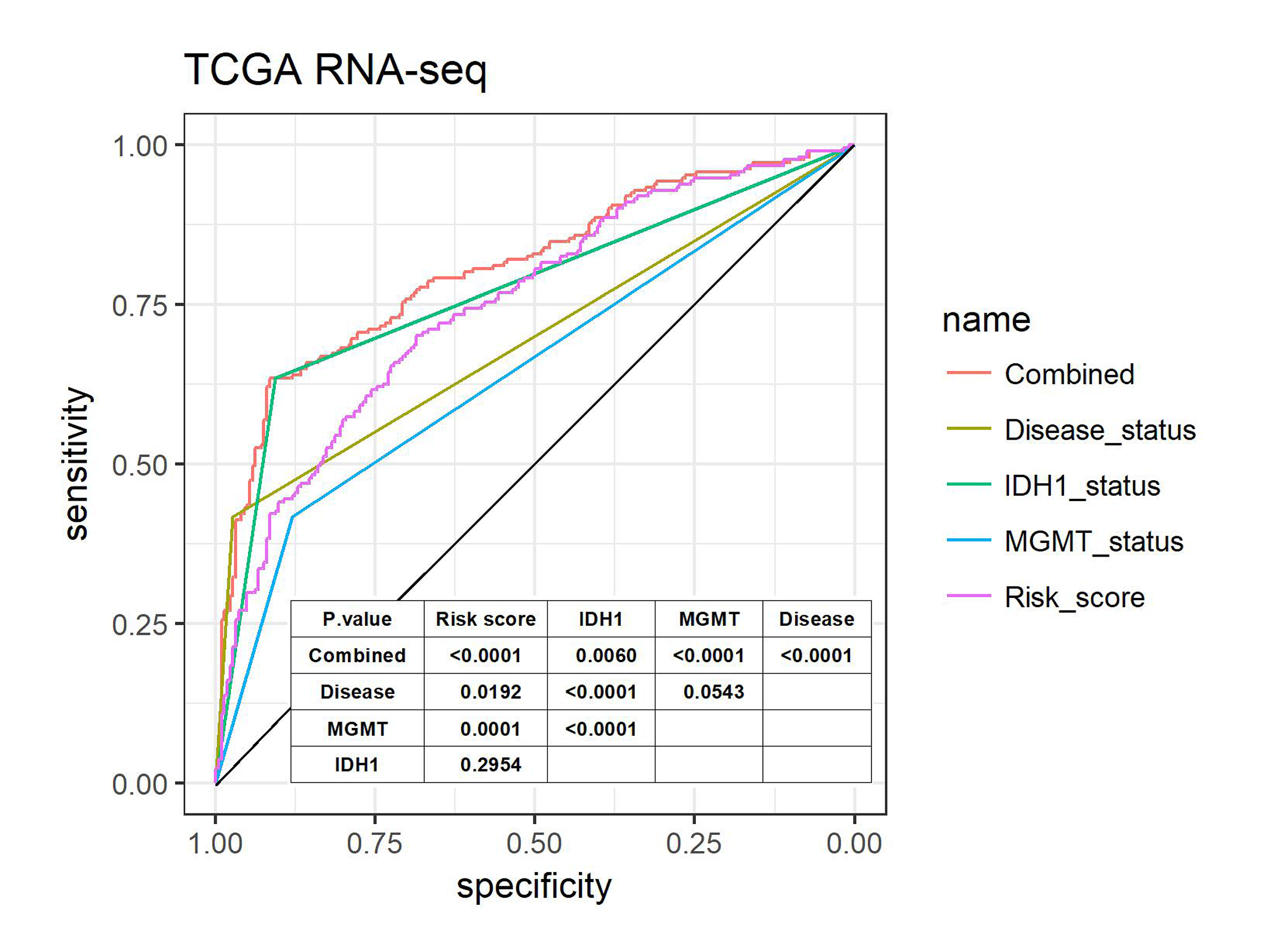


**Supplementary Figure 5. ROC analysis regarding the validation cohort TCGA RNA-seq.**

The ROC result showed the AUC of disease status (GBM vs LGG, AUC=0.6953), IDH1 mutation status (wild type vs mutant, AUC=0.7711), MGMT methylation status (unmethylated vs methylated, AUC=0.6488) and the risk score (as continuous variable, AUC=0.7490). The risk score outperformed disease status (P=0.0192) and MGMT methylation status (P=0.0001) while showed no significant difference compared with IDH1 mutation status (P=0.2954). A significant higher AUC (combined, AUC=0.8072) was achieved when applied fitting generalized linear model to the risk score (P<0.0001) and IDH1 mutation status (P=0.0060).


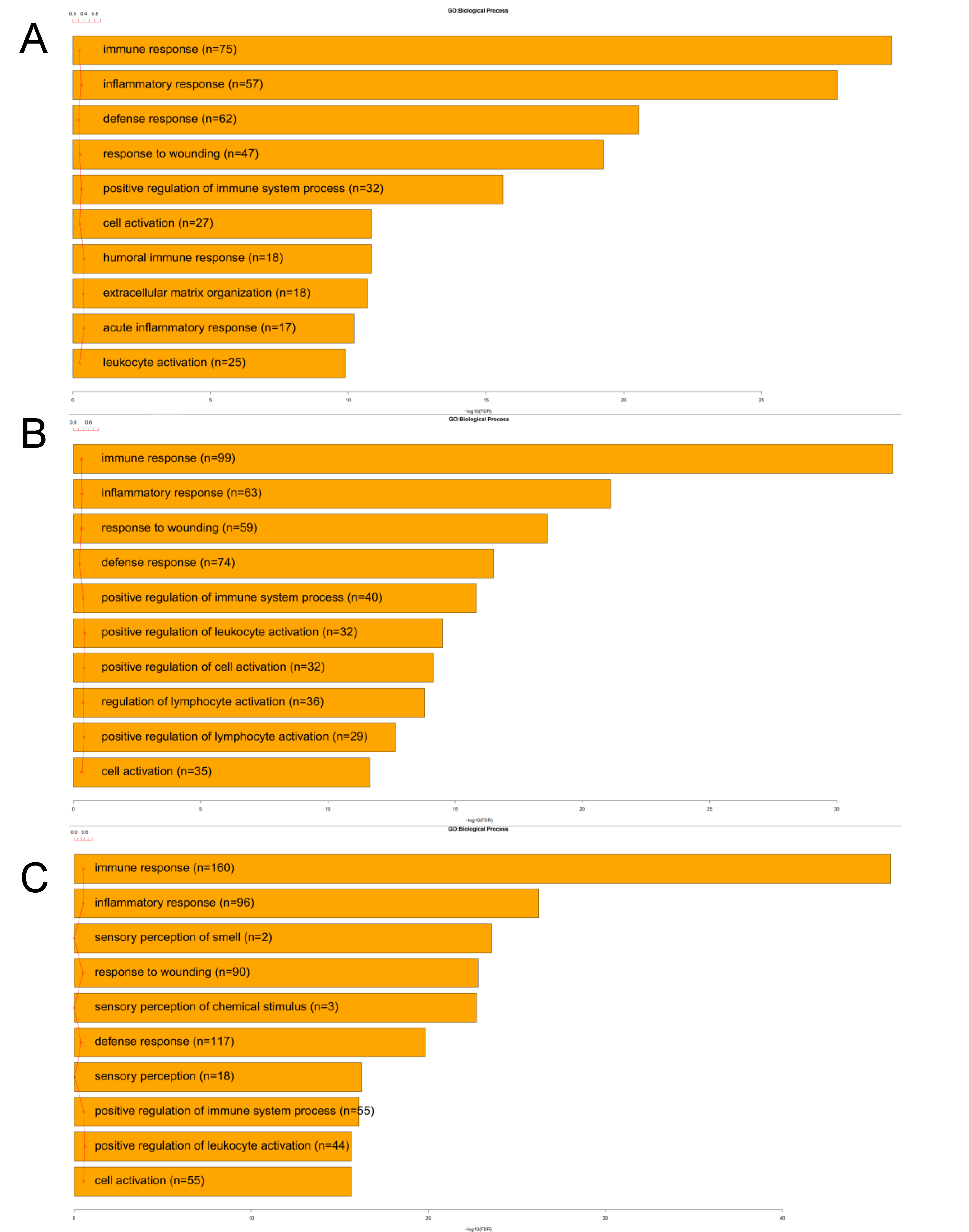
**Supplementary Figure 6. GO** **annotation of the TF signature in the other three validation cohort. (A)** Top 10 GO annotation in the GSE16011 cohort. **(B)** Top 10 GO annotation in the CGGA SEQ cohort. **(A)** Top 10 GO annotation in the TCGA663 SEQ cohort.

**
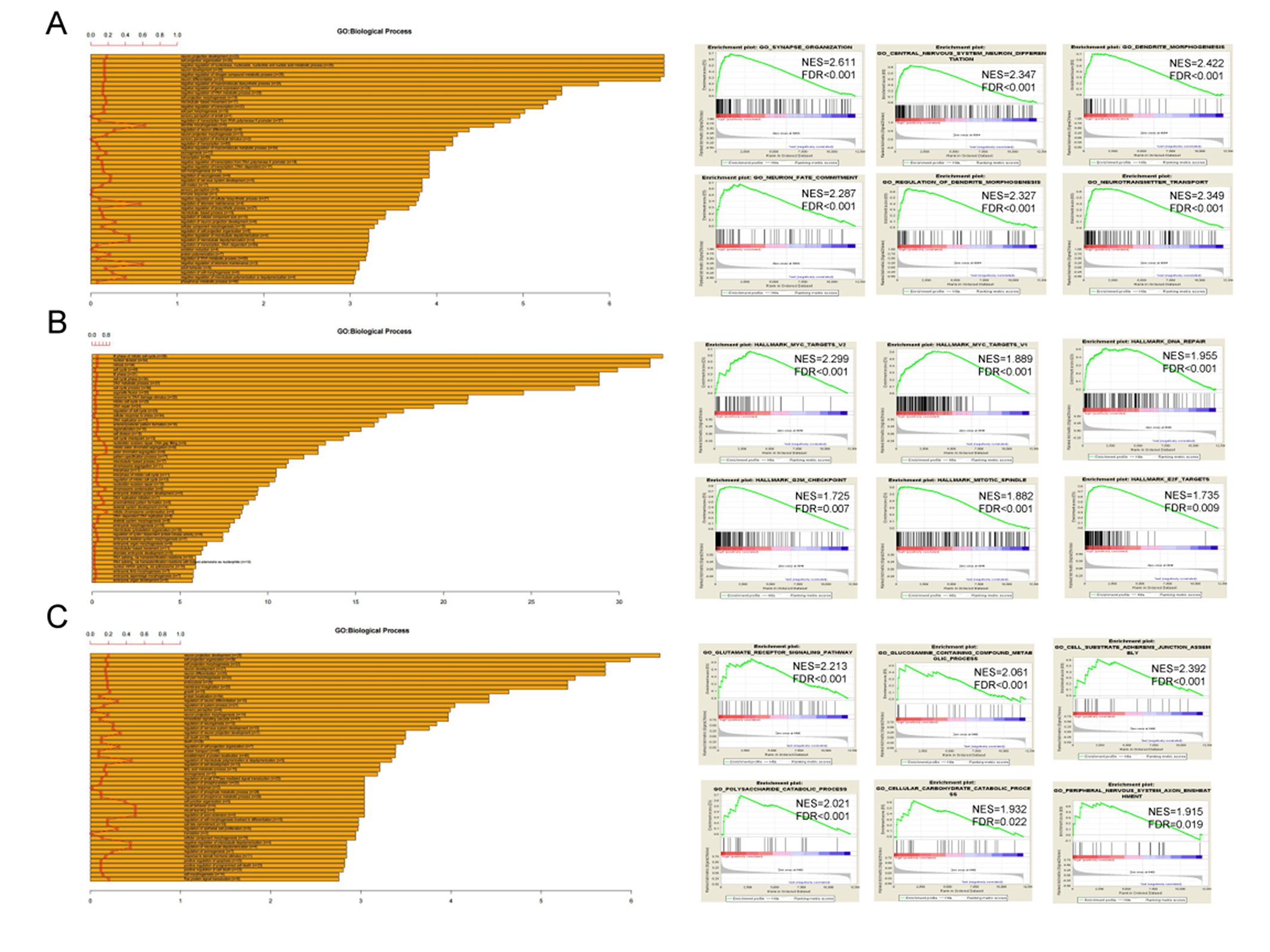
 Supplementary Figure 7. Function annotation of the other three clusters of TF gene sets in the training cohort. (A)** GO and GSEA analyses based on the cluster 2 TF gene set indicated that the gene set was associated with neuron development. **(B)** GO and GSEA analyses based on the cluster 3 TF gene set indicated that the gene set was associated with cell cycle. **(C)** GO and GSEA analyses based on the cluster 4 TF gene set indicated that the gene set was associated with metabolic and neuron morphogenesis.

**
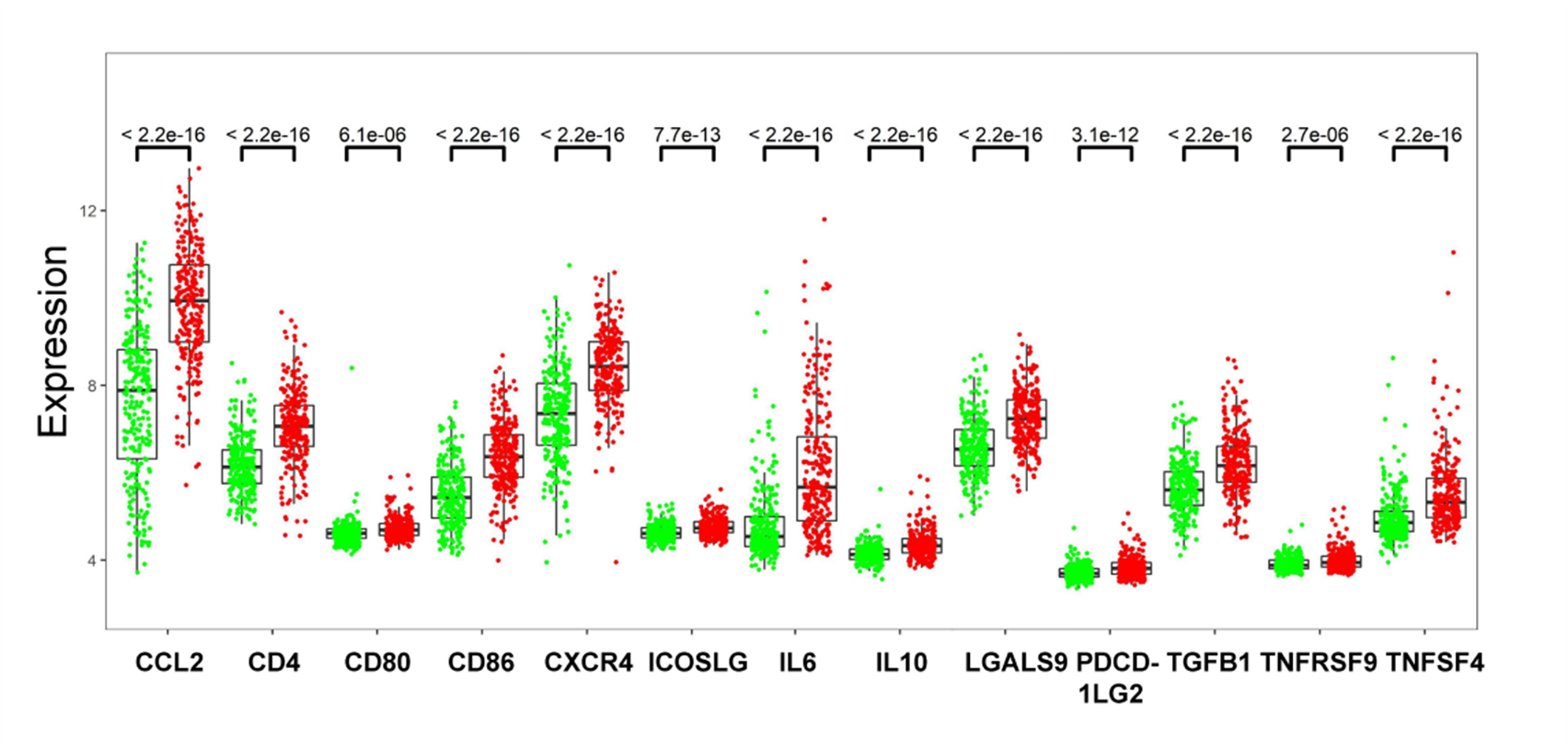
 Supplementary Figure 8. Different expression pattern of the immune checkpoint genes in the training cohort.** The immune checkpoint genes (CLL2, CD4, CD80, CD86, CXCR4, ICOSLG, IL6, IL10, LGALS9, PDCD1LG2, TGFB1, TNFRSF9 and TNFSF4) were found to be more expressed in high risk score group than low risk score group in the training cohort.

**Supplementary Table 1** Clinical characteristics of the risk score in the training cohort and validation cohort.

| Patient characteristics | Training cohort (TCGA Microarray) | | | Validation cohort (GSE16011) | | |
| --- | --- | --- | --- | --- | --- | --- |
|  | Risk Score  Low  (n=262) | Risk Score  High  (n=263) | P value | Risk Score  Low  (n=77) | Risk Score  High  (n=78) | P value |
| Age (year) |  |  | 0.7611 |  |  | **0.0243** |
| <60 | 134 | 139 |  | 55 | 41 |  |
| ≥60 | 128 | 124 |  | 22 | 37 |  |
| Gender |  |  | 0.1980 |  |  | **0.0085** |
| Male | 152 | 168 |  | 44 | 61 |  |
| Female | 110 | 95 |  | 33 | 17 |  |
| Grade |  |  | - |  |  | - |
| IV | 262 | 263 |  | 77 | 78 |  |
| KPS |  |  | 0.8143 |  |  |  |
| ≥80 | 151 | 142 |  |  |  |  |
| <80 | 49 | 50 |  |  |  |  |
| IDH1 |  |  | **<0.0001** |  |  | 0.2655 |
| Mutant | 29 | 5 |  | 20 | 13 |  |
| Wild type | 169 | 207 |  | 43 | 48 |  |
| MGMT |  |  | **0.0176** |  |  |  |
| Methylated | 88 | 69 |  |  |  |  |
| Unmethylated | 82 | 110 |  |  |  |  |
| Transcriptional subtype |  |  | **<0.0001** |  |  | **<0.0001** |
| Neural | 40 | 21 |  | 9 | 9 |  |
| Proneural | 83 | 24 |  | 33 | 5 |  |
| Classical | 98 | 57 |  | 34 | 36 |  |
| Mesenchymal | 16 | 157 |  | 1 | 28 |  |

**Continued on next page**

| Patient characteristics | Validation cohort (CGGA RNA-seq) | | | Validation cohort (TCGA RNA-seq) | | |
| --- | --- | --- | --- | --- | --- | --- |
|  | Risk Score  Low  (n=58) | Risk Score  High  (n=59) | P value | Risk Score  Low  (n=331) | Risk Score  High  (n=332) | P value |
| Age (year) |  |  | 0.1856 |  |  | **<0.0001** |
| <60 | 54 | 42 |  | 291 | 220 |  |
| ≥60 | 4 | 17 |  | 40 | 112 |  |
| Gender |  |  | 0.5148 |  |  | **0.0136** |
| Male | 36 | 41 |  | 174 | 207 |  |
| Female | 22 | 18 |  | 157 | 125 |  |
| Grade |  |  | - |  |  | **<0.0001** |
| II |  |  |  | 157 | 57 |  |
| III |  |  |  | 123 | 116 |  |
| IV | 58 | 59 |  | 11 | 142 |  |
| KPS |  |  |  |  |  | 0.1063 |
| ≥80 |  |  |  | 148 | 159 |  |
| <80 |  |  |  | 24 | 42 |  |
| IDH1 |  |  | **<0.0001** |  |  | **<0.0001** |
| Mutant | 26 | 3 |  | 299 | 124 |  |
| Wild type | 32 | 56 |  | 31 | 202 |  |
| MGMT |  |  |  |  |  | **<0.0001** |
| Methylated |  |  |  | 293 | 180 |  |
| Unmethylated |  |  |  | 37 | 122 |  |
| Transcriptional subtype |  |  | **<0.0001** |  |  | **<0.0001** |
| Neural | 10 | 0 |  | 81 | 29 |  |
| Proneural | 23 | 2 |  | 174 | 60 |  |
| Classical | 21 | 21 |  | 10 | 76 |  |
| Mesenchymal | 4 | 36 |  | 0 | 96 |  |

P value was calculated by Chi-squared or Fisher’s exact test depending on patient counts in each group.

**Supplementary Table 2** Correlation and prognostic value of each immune cell in the training cohort.

| Immune cell | Log-rank test | Correlation with risk score (Pearson correlation) | |
| --- | --- | --- | --- |
|  | P | r value | P |
| Macrophages | **<0.001** | 0.817 | **<0.001** |
| iDC | 0.447 | 0.636 | **<0.001** |
| Neutrophils | 0.787 | 0.582 | **<0.001** |
| NK_CD56dim | 0.111 | 0.496 | **<0.001** |
| Th17 | 0.130 | 0.425 | **<0.001** |
| aDC | **0.024** | 0.386 | **<0.001** |
| Cytotoxic | 0.413 | 0.356 | **<0.001** |
| Mast | 0.541 | 0.346 | **<0.001** |
| Eosinophils | **0.004** | 0.323 | **<0.001** |
| Th1 | **0.024** | 0.308 | **<0.001** |
| T cell | 0.264 | 0.274 | **<0.001** |
| DC | 0.431 | 0.236 | **<0.001** |
| B | 0.601 | 0.078 | 0.072 |
| NK | 0.825 | -0.027 | 0.537 |
| TFH | 0.859 | -0.083 | 0.059 |
| Tgd | 0.162 | -0.095 | **0.030** |
| TReg | 0.293 | -0.106 | **0.015** |
| NK_CD56bright | 0.658 | -0.107 | **0.014** |
| Tcm | 0.729 | -0.130 | **0.003** |
| Tem | 0.396 | -0.181 | **<0.001** |
| CD8 | 0.076 | -0.193 | **<0.001** |
| T helper | **0.028** | -0.248 | **<0.001** |
| Th2 | 0.776 | -0.298 | **<0.001** |
